# Assessment of intensity, prevalence and duration of everyday activities in Swiss school children: a cross-sectional analysis of accelerometer and diary data

**DOI:** 10.1186/1479-5868-6-50

**Published:** 2009-08-05

**Authors:** Bettina Bringolf-Isler, Leticia Grize, Urs Mäder, Nicole Ruch, Felix H Sennhauser, Charlotte Braun-Fahrländer

**Affiliations:** 1 Institute of Social and Preventive Medicine, Steinengraben 49, 4051 Basel, Switzerland; 2 Federal Institute of Sports, 2532 Magglingen, Switzerland; 3 University Children's Hospital, Steinwiesstrasse 75, 8032 Zürich Switzerland

## Abstract

**Background:**

Appropriately measuring habitual physical activity (PA) in children is a major challenge. Questionnaires and accelerometers are the most widely used instruments but both have well-known limitations. The aims of this study were to determine activity type/mode and to quantify intensity and duration of children's everyday PA by combining information of a time activity diary with accelerometer measurements and to assess differences by gender and age.

**Methods:**

School children (n = 189) aged 6/7 years, 9/10 years and 13/14 years wore accelerometers during one week in winter 2004 and one in summer 2005. Simultaneously, they completed a newly developed time-activity diary during 4 days per week recording different activities performed during each 15 min interval. For each specific activity, the mean intensity (accelerometer counts/min), mean duration per day (min/d) and proportion of involved children were calculated using linear regression models.

**Results:**

For the full range of activities, boys accumulated more mean counts/min than girls. Adolescents spent more time in high intensity sports activities than younger children (p < 0.001) but this increase was compensated by a reduction in time spent playing vigorously (p = 0.04). In addition, adolescents spent significantly more time in sedentary activities (p < 0.001) and accumulated less counts/min during these activities than younger children (p = 0.007). Among moderate to vigorous activities, children spent most time with vigorous play (43 min/day) and active transportation (56 min/day).

**Conclusion:**

The combination of accelerometers and time activity diaries provides insight into age and gender related differences in PA. This information is warranted to efficiently guide and evaluate PA promotion.

## Background

Childhood overweight and obesity are increasing in many countries including Switzerland [1] and there is growing concern that decreasing levels of physical activity (PA) may contribute to this development. Still, appropriately measuring PA in children is a major challenge. Questionnaires and accelerometer measurements are the most widely used instruments [2,3]. Self- or proxy reports provide information about mode/type and duration of PA but show limited validity in assessing PA levels and are susceptible to reporting bias by social desirability [4]. On the other hand, accelerometer measurements provide valid overall estimates of intensity of PA [5,6]. Nevertheless, they neither determine which activities contribute most or least to PA in children nor the variation in the type and duration of habitual activities over time. Yet, this information is of great importance for public health authorities in order to efficiently guide PA promotion and to evaluate changes in PA levels over time, by gender or with age. So far, only few studies have combined accelerometer measurements with self-report data, taking advantage of the unique pieces of information that each instrument provides [7-9]. However, the self-report part in the above cited studies is based on questionnaires or activity logs but not on continuous physical activity records which, based on the exact time specification, can be compared minute by minute to accelerometer outputs. This allows to calculate the mean intensity for each activity and the summed time spent in it and thus to evaluate which activity contributes most or least to physical activity by gender and age.

In the framework of a pilot study for a monitoring programme of PA levels in Swiss school children, we developed a time-activity diary including a list of 21 typical everyday activities and asked parents (or adolescents) to allocate to each of 15-minute intervals of the child's (or their) day a specified activity. Concomitant to completion of the diary, the children wore an accelerometer device. The aims of this study were to determine activity type/mode and to quantify intensity and duration of children's everyday PA by combining information of a time-activity diary with accelerometer measurements and to assess differences by gender and age.

## Methods

### Sample

**Figure 1:**
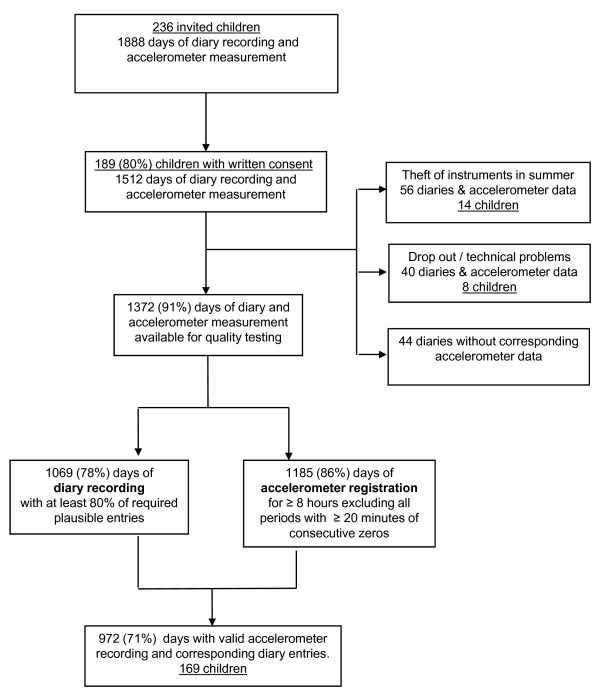
**Schematic of the study design**.

Participants were part of a larger cross-sectional study [10] which included three age groups of children (kindergarten/1^st ^grade, 4^th ^grade and 8^th ^grade) living in three communities (Bern, Biel-Bienne and Payerne). For the present study, children in a random sample of 19 school-classes (at least two classes per grade and per community) were invited to wear an accelerometer device and to complete a time-activity diary. A participation flow chart is shown in figure 1. Personal and social characteristics of the children in invited classes did not differ significantly from those in non-invited classes. The study protocol was approved by the ethics committee of the University of Bern, and parents gave written consent.

### Accelerometer

Objective assessment of PA was obtained over two seven day periods, one in winter 2004 and one in spring/summer 2005, using Actigraph accelerometers (Model AM7164, formerly Computer Science and Applications (CSA), now Manufacturing Technology Inc. (MTI), Fort Walton Beach, FL). The device measures the change in body position taking 40 measurements per second and integrates acceleration signals continuously (epoch time 1 minute). The summed values (activity counts) were stored in the device memory and downloaded to a computer. Participants were instructed to wear the accelerometer rigidly fixed at the waist with a belt. It was not worn during sleeping hours, bathing or other water activities.

### Diary (physical activity record)

The diary, highly structured, required the continuous record of activities performed during each 15-minutes intervals between 6:00 and 22:00 h and each hour between 23:00 and 6:00 h. Its format has been adapted from an existing diary used for the sleeping and feeding time assessment in babies [11]. An English version of the time-activity diary is presented in Additional file 1. It has been suggested that physical activity records provide more detail about the type, intensity and patterns of activity completed during the day than physical activity logs [12]. In addition, the given continuous timeline facilitates the recording of time spent in a specific activity, thus reducing potential recall bias. Activities, which were expected to represent low intensity levels [13] included: watching TV, sitting at a computer, playing a music instrument, reading, attending class, performing homework, playing quietly, eating, travelling by car, travelling by public transport and going out. Activities expected to be of moderate intensity [13] included: cycling, walking, attending recess, moderately intense playing indoors and outdoors. Finally, activities assumed to represent vigorous intensities [13] were: attending physical education (PE) classes, sports training indoors and outdoors and vigorously playing indoors and outdoors. A first selection of activities based on existing diaries [13]has been pre-tested in two primary school classes with different social background and in 10 adolescents. After an interview with these children, the list of activities was adopted to capture activities, which differ among age groups. Less specific activities such as attending recess or attending PE-class describe contexts where children are expected to be active. "Attending recess" means the time (15 to 30 minutes) between lessons spent in the school recreation area. As in the Swiss school system the number of lessons per week increases with age, also time attending recess increases. PE classes are integrated in the class schedule. By law, all school children (but not those in the kindergarten) receive three 45 min PE classes per week. In contrast to training (which in Switzerland is organized completely independent of the school activities) PE classes do not focus on a specific sport.

Students (8^th ^grade) or parents (kindergarten, 1^st ^grade and 4^th ^grade) were instructed to mark at least one activity for any given 15 minutes interval. The diary also allowed marking sleeping time, an activity difficult to classify and the removal of the accelerometer. The diary was completed during two week days and a weekend, concomitant to the accelerometer recording periods.

### Questionnaire

Information on children's age, sex, nationality, parental education, weight and height and leisure time habits were extracted from the main survey parents' completed questionnaire. Parents filled in the questionnaire two months before the accelerometer measurements and completitions of diaries.

### Meteorological data

For each community and time periods of accelerometer and diary recording, daily meteorological data were provided by MeteoSwiss [14]. Measurements included mean, maximum and minimum temperatures (range -9.5 to 17.1°C), atmospheric pressure (945–971 hPa), relative humidity (57–97%), sun radiation (10–318 watt/km^2^), sum of sunshine duration (0–798 min), and sum of precipitation (0–18.2 mm).

### Procedure

The study was conducted during the school year 2004/2005 and was organized within the framework of the School Health Services. Children and their families received written instructions on how to use the accelerometer and how to fill in the diary. In addition, trained staff instructed the children at school. The 13–14 years olds completed the diary on their own, the younger ones with their parents.

### Data processing

The completeness and plausibility of all diary entries and MTI outputs were checked. If two or more activities indicated during the same 15 min interval were logically not compatible (e.g. sleeping and eating), they were set to missing. If data were missing between 23:00 and 6:00 h, the activity was considered to be 'sleeping'. Diaries having 80% of the 15 minutes blocks filled in with logically plausible entries were considered valid for analyses. If two or more activities were indicated the more intense was considered.

The length of an accelerometer-recording day was individually defined. A day started if 10 consecutive minutes had at least one value of 800 counts/min and not more than one value with zero counts/min. The individual day stopped if after the measurement, which was considered to be the last of the day, there was an inactive period of four consecutive hours. MTI outputs equal to zero for more than 20 continuous minutes were excluded, assuming that the device was not worn during this period. Special attention was given to artefacts in accelerometer measures, which can occur if accelerometers are hit resulting in extremely high counts/min. Therefore, all values above 30,000 counts/min were substituted by the mean counts/min of the respective age group. Only days with at least 8 hours of registration were considered valid for the analyses.

For the analysis of the intensity of single activities valid diary and accelerometer days were matched by exact point in time. The information about the duration of specific activities is based on the diary.

### Validation of the diary

Total time spent in moderate to vigorous physical activity (MVPA) assessed by the time-activity diary was validated using accelerometer measurements. Metabolic equivalents (MET) based on accelerometer counts were calculated using the cut off levels of Freedson/Trost [15]. MET levels equal or above 3 were defined as MVPA. Spearman correlation between total time spent in MVPA per day based on an a priori classification of specific activities in the diary and total time with MET levels ≥ 3 was moderate (r = 0.52) and statistically significant (p ≤ 0.001).

### Statistical Analysis

All analyses were conducted with STATA 9.0 [16]. Univariate logistic regression models were used to examine differences in personal and social factors between participants and non-participants.

Mixed linear regression analysis was used to determine the association between accelerometer counts/min and socio-demographic and environmental characteristics. The models included age, sex, maternal education, nationality, the day of the week (weekday/weekend), season (winter/summer), mean daily temperature, the sum of precipitation and a random effect for subject.

For each child, the mean intensity for every given activity (counts/min) over all 15 minutes were calculated. Differences in intensity of a specific activity between age groups and between gender as well as interactions between age and gender were assessed by linear regression analysis.

Mixed linear regression models with a random effect for subject were generated to evaluate age and gender differences in mean duration spent in a given activity while simultaneously taking into account the effect of the day of the week, the season and meteorological factors. As the distribution of the residuals was skewed, standard errors of regression estimates were determined using a bootstrap (with 1000 replications) [17]. The final multivariate model to estimate activity duration (min/day) included the following variables: sex, age, maternal education, sum of precipitation above a threshold of 4 mm, and maximum temperature.

## Results

### Study population

Of the189 participants, 164 completed diaries in both seasons, 3 only in summer and 22 only in winter. If parents were non-Swiss (53.9% vs. 85.7% valid diaries) or less educated (62% vs. 82%), more diaries had to be excluded because of low quality.

### Sociodemographic characteristics

Sociodemographic and environmental characteristics and their association with mean counts/min are shown in table 1. Mean counts/min decreased significantly with age and were significantly lower in girls. The small number of overweight children in this sample (n = 12) did not allow the evaluation of overweight related differences in activity levels. In addition, PA increased with temperature (7.8 counts/min per 1°C increase) and decreased with the sum of precipitation (-6.2 counts/min per 1 mm of precipitation over a threshold of 4 mm/day).

**Table 1:** Socioeconomic and environmental characteristics and their association with accelerometer counts/min

**Social and environmental factors**	**Total n (%)**	**Accelerometer counts/min: adjusted^§ ^mean (95%CI)**
Grade (Age)		
1st (6/7 years) (reference group = ref.)	47 (28)	751 (712–790)
4th (9/10 years)	60 (36)	662 (626–698)**
8th (13/14 years)	62 (38)	546 (508–583)***
		
Sex		
Boys (ref.)	81 (48)	737 (706–767)
Girls	88 (52)	569 (539–599)***
		
Education Mother		
Low (ref.)	22 (14)	636 (584–717)
Middle	80 (49)	672 (638–698)
High	61 (37)	633 (599–668)
		
Nationality		
Swiss (ref.)	129 (78)	649 (624–675)
Non Swiss	37 (22)	661 (602–720)
		
Day of the week		
Weekend (ref.)	414 (44)	631 (603–659)
Weekday	532 (56)	670 (645–696)*
		
Season		
Winter (ref.)	502 (53)	638 (604–671)
Summer	444 (47)	670 (634–706)

^§ ^Mutually adjusted estimates based on multivariate regression models including all variables presented in the table as well as mean daily temperature, sum of precipitation, and a random effect for subject

* p ≤ 0.05, **p ≤ 0.01, *** p ≤ 0.001; compared to reference group

### Intensity

Intensities varied significantly among activities. Highest raw mean counts/min were achieved when children were involved in sports training outdoors (1513 counts/min) and lowest when playing a music instrument (392 counts/min). Watching television (407 counts/min) yielded similarly low counts/min as sedentary activities such as reading (467 counts/min).

**Figure 2:**
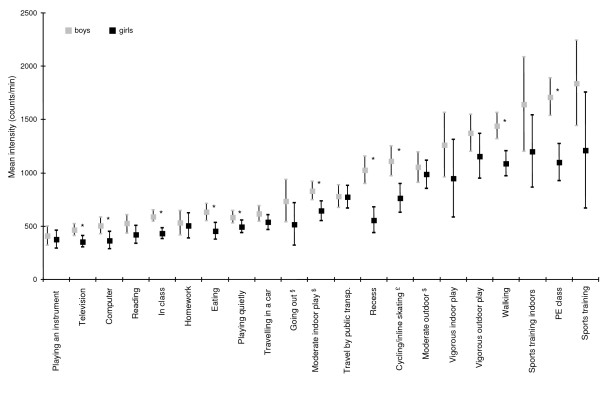
**Age adjusted intensities for specific activities by gender**. (* p < 0.05; ^§^only assessed in 8th graders; ^$^only assessed in the two younger age groups; ^£^cycling not appropriately measured by accelerometer). Some inaccuracy estimating the intensity of each activity may have occurred because the start and end of the activities were based on the activity log.

Figure 2 gives the adjusted mean counts/min for each specific activity by gender. For all activities, boys' mean counts/min clearly exceeded those of girls. These differences were statistically significant for 10 out of 21 activities. The variation of intensity was smaller in sedentary activities and higher in more active ones as well as such, that are mainly a description of the context (as recess). There was no interaction between age and gender with respect to intensity. The three self-reported intensity levels of playing in the diary corresponded to accumulated accelerometer counts/min in both sexes. Outdoors activities were associated with higher counts/min (1017 (935–1080) and 1259 (971–1586) for playing moderately and vigorously outdoors) than the corresponding indoor activity (734 (582–867) and 1097 (718–1629) for playing moderately and vigorously indoors).

**Figure 3:**
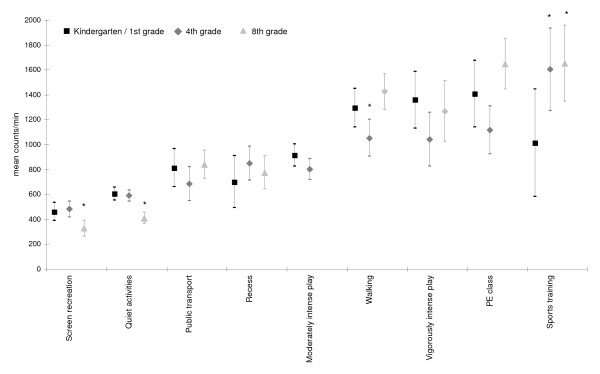
**Gender adjusted intensities for specific activities by age group**. Screen recreation: watching television andsitting at a computer. Quiet activities: playing a music instrument, reading, in class, homework, eating, playing quietly and travelling by car. Moderately intense play: moderately intense play indoors and moderately intense play outdoors. Vigorously intense play: vigorously intense play indoors and vigorously intense play outdoors. Sport training: indoors sports training andoutdoors sports training. Cycling was excluded, as it can't be measured by accelerometer in an appropriate mode.

Figure 3 displays adjusted mean intensities by age group. Individual activities were summarized into broader categories (see legend figure 3). For screen recreation and the other quiet activities, 8^th ^graders accumulated significantly (p < 0.001) less mean counts/min when compared to the youngest age group. However, during sports training, they achieved significantly higher counts/min (p = 0.002). In the two younger age groups, mean counts/min accumulated during walking and vigorously playing indoors/outdoors were similar to those achieved during physical education classes at school. In the youngest age group walking (1296 counts/min) and vigorously playing (1358.2 counts/min) yielded higher mean counts/min than sports training indoors (894 counts/min) or outdoors (1031 counts/min).

### Duration and prevalence

Next, the mean time spent in a given activity (table 2) and the prevalence of children engaged in the respective activity were evaluated. Most time was spent with sedentary activities. Among moderate to vigorous activities, children spent most time in active transportation or playing outdoors. Only 37% of the children attended sports training during the four diary reporting days, hence, mean duration of sports training over all children was very short.

**Table 2:** Adjusted^§ ^mean duration of specific activities by age group

		**Activity duration in min/day**				
**Activity**		**All Children**	**Kindergarten/1^st ^grade (6/7 years old)**	**4^th ^grade (9/10 years old)**	**8^th ^grade (9/10 years old)**	***P*^$^**
		Mean^§ ^(95% CI)	Mean^§ ^(95% CI)	Mean^§ ^(95% CI)	Mean^§ ^(95% CI)	
Screen recreation		104.4 (98.8–110.1)	71.0 (62.3–79.7)	105.9 (96.9–115.0)	135.8 (123.2–148.5)	< 0.001
	Television	76.2 (71.2–81.0)	56.8 (49.1–64.5)	77.0 (68.5–85.5)	94.3 (83.1–105.5)	< 0.001
	Computer	28.2 (25.3–31.1)	14.2 (10.7–17.6)	28.8 (24.2–33.4)	41.4 (35.0–47.7)	< 0.001
Any other quiet activity		391.7 (384.0–399.3)	364.1 (350.3–378.0)	391.6 (378.9–404.2)	418.9 (403.9–434.0)	0.002
	Leisure time activities^£^	238.4 (231.2–245.7)	249.9 (237.1–262.7)	247.3 (235.6–259.1)	216.9 (203.9–230.0)	< 0.001
	Attending school/homework^#^	261.2 (254.0–268.5)	200.2 (189.3–211.1)	256.1 (244.9–267.2)	327.5 (313.7–341.3)	< 0.001
Active Transportation		56.0 (52.3–59.7)	54.9 (48.7–61.1)	51.1 (45.4–56.7)	62.52 (54.8–70.3)	0.029^&^
	Walking	40.6 (37.3–42.8)	41.9 (36.5–47.4)	33.5 (28.6–38.4)	47.2 (40.1–54.4)	0.198
	Cycling/inline skating	15.4 (13.3–17.6)	13.0 (9.6–16.4)	17.6 (13.6–21.5)	15.4 (11.4–19.3)	0.394
Recess^#^		25.1 (22.6–27.7)	9.9 (7.2–12.7)	27.5 (23.6–31.4)	37.6 (31.0–44.3)	< 0.001
Vigorously intense unstructured play		42.9 (38.0–47.8)	42.7 (34.7–50.7)	49.6 (41.4–57.7)	35.7 (27.4–44.0)	0.035^$^
	Indoors	9.7 (7.5–11.8)	14.1 (9.7–18.6)	7.1 (3.7–10.4)	8.1 (4.5–11.8	0.037
	Outdoors	33.3 (28.9–37.7)	28.5 (21.6–35.5)	42.5 (34.4–50.6)	27.6 (19.5–35.7)	0.853
Attending PE° classes^#^		27.4 (23.1–31.7)	21.2 (13.2–29.2)	24.8 (19.3–30.2)	36.3 (28.5–44.2)	0.008
Sports training		10.7 (8.3–13.0)	4.7 (2.3–7.1)	10.8 (6.7–15.0)	16.5 (11.3–21.7)	< 0.001

^§ ^Estimates adjusted for sex, maternal education, season, day of the week, mean daily temperature, sum of precipitation and subject (as random effect)

^$ ^Test for trend

^£ ^Leisure time activities: playing a music instrument, reading, quietly playing and riding a car

^# ^Duration calculated excluding weekends

^&^8^th ^graders compared to kindergarten/1^st ^graders and 4^th ^graders

° Physical education

Boys spent significantly more time sitting at a computer than girls (adj. mean duration (95% CI): 38.3 (33.7–42.8) min/day and 18.0 (14.5–21.4) min/day, p < 0.001, respectively). Girls, on the other hand reported a longer duration of playing quietly than boys (102.8 (95.4–110.3) min/day and 82.7 (75.3–90.1) min/day, p < 0.001, respectively). Significantly more boys (85.2%) reported vigorously playing outdoors compared to girls (59.1%) and they spent more time with this activity than girls (47.9 (40.1–55.8) min/d and 18.5 (14.1–23.0) min/day p < 0.001, respectively).

With increasing age, the duration of screen recreation and school related quiet activities increased significantly and there was a shift from unorganized PA (vigorously playing) to organized PA (PE classes or attending sports) (table 2). Furthermore, eighth graders spent significantly more time in active modes of transportation and in attending recess at school.

With decreasing level of maternal education, children's time watching TV increased (adj. mean duration (95% CI): 66 (60–72) min/day, 79 (71 -88) min/day, and 98 (80–115) min/day, respectively for low, middle and high levels of maternal education) whereas time spent reading books decreased (31 (26–35) min/day, 23 (19–26) min/day and, 17 (11–24) min/day, respectively). Further, children of mothers with low educational levels spent less time playing vigorously intense compared to children of mothers with high educational levels (adj. mean duration (95% CI): 45 (36–53) min/day, 45(38–52) min/day, and 27 (16–37) min/day, respectively, for high, middle and low level of maternal education). Yet, mean counts/min did not differ significantly by maternal education (table 1).

The duration of many activities was influenced by season, the day of the week and meteorological parameters. There was a significant decrease in time spent vigorously playing outdoors (-1.7 (-2.7 to -0.7) min/day per 1 mm rainfall, p < 0.001), biking (-0.88 (-1.3 to -0.5) min/day per mm rainfall, p < 0.001) and going out (-2.4(-4.6 to -0.2) min/day per mm rainfall (activity assessed in adolescents only), p < 0.03) with the sum of precipitation whereas activities typically performed indoors such as sitting at a computer increased (1.0 min/day per mm rainfall, p = 0.037). In summer, children spent significantly more time playing vigorously outdoors (42.3 (32.9–51.7) min/day) than in winter (25.2 (17.0–33.4) min/day; p = 0.02). On weekends, significantly more time was spent in quiet leisure activities (284.4 (271.0–297.9) min/day and 191 (182.5–200.4) min/day for weekends and weekdays respectively, p = 0.001), and in vigorous play (54.0 (44.8–63.3) min/day and 34.2 (28.4–39.4) min/day for weekends and weekdays respectively, p ≤ 0.001) than on weekdays.

## Discussion

The present study combined objectively measured accelerometer data with a detailed assessment of the exact time point and the duration of single activities in a time-activity diary (physical activity record), allowing precisely estimated intensity and duration of specific activities. This combination enabled the unique pieces of information that each instrument provides to be integrated and showed significant differences in intensity and duration of specific activities by gender and age. Compared with physical activity logs [13] the registration of each activity along a time-line provided more in-depth insight into physical activity patterns of school-aged children.

In line with other research, the present study found girls to be less active than boys [7,18-20]. It has previously been reported that during PE classes [9,21] and during recess [22], boys accumulate more counts/min than girls, yet, the results of the present study indicated that girls collected systematically less counts/min for the full range of everyday activities. However, the observed gender difference in PA was also due to differences in activity patterns such as girls spending significantly less time playing vigorously. In contrast to a previous study [7], the present study did not observe significant gender differences in the prevalence or duration of structured sports or PE classes.

The decrease in PA with age followed a more complex pattern. On one hand, more adolescents were engaged in high intensity activities such as structured sports, spending significantly more time in these activities than younger children. On the other hand, younger children spent more time playing vigorously than adolescents. In addition, adolescents spent significantly more time in sedentary activities but accumulated less accelerometer counts/min during sedentary activities than younger children. The combination of these factors resulted in significantly less mean counts/min for adolescents.

Younger children accumulated high count values predominantly during unstructured moderate and vigorous intense play whereas structured sports activities were less important. This is consistent with previous studies [18,23], reporting that time spent playing outdoors was an important contributor to PA in children

Walking was another important activity contributing to PA in all age groups. The present study underlines the importance of active commuting for PA levels of children [10,24,25]. In the Swiss context and most likely in other European countries, it would thus not be sufficient to only focus on playing outdoors to assess PA in younger children as has been suggested by Burdette et al. [23].

The detailed assessment of different activities with the time-activity diary also provided insight into subtle activity differences related to the educational background of the child's mother. Total mean counts/d did not vary significantly with maternal education, but the type of passive activities (TV and reading books) varied. Watching television is not only problematic because of its low intensity but also because of its association with overweight [26,27]. This association might not only be due to the lack of PA, but also result from poorer eating habits [28].

Consistent with previous findings [29,30], the present analyses also illustrate the importance of meteorological conditions for the assessment of different outdoor activities. Moreover, it is noteworthy that self-reported intensities of playing corresponded well with accelerometer measurements and differentiating between playing "indoors" and "outdoors" added useful information to the activity assessment.

Compared to previous studies [7,8] the combination of the time-activity diary with simultaneous accelerometer measurements provided precisely estimated intensity and duration of a large range of everyday activities and allowed to detect systematic differences in intensity between gender or between age-groups. In contrast to PA questionnaires and to physical activity logs, the fixed 24-hour timeline of our time-activity diary facilitated the assessment of specific activities by the parents and their children. Repeating such accelerometer measurements along with a time-activity diary as part of a monitoring programme will allow to determine changes in physical activity pattern and the relative contribution of specific activities to overall childhood physical activity levels over time.

Although the use of the time-activity diary in the present study provided valuable information about children's activity pattern, several limitations became apparent. First, it has to be acknowledged that the proportion of good quality filled diaries depended on maternal education and nationality, thus leading to an under-representation of lower social classes and non-Swiss populations limiting the generalizability of our findings. Second, for future use of the diary, it is suggested to assess exactly the same activities for all children, as comparisons across age groups are otherwise limited. Third, the sample size was rather small for stratified analyses. Fourth, attending recess or attending PE class are a description of a context which is expected to be active but we did not collect exact data on the type of activity for this period. Last, the documentation of only four days of the week potentially underestimates activities like sports training. In the parents' questionnaire, 59% of the children were reported to attend sports training whereas only 37% indicated this activity during the 4 days of diary recording.

## Conclusion

The combination of accelerometer and time activity diaries allowed the precise quantification of both the intensity and the duration of children's everyday PA and provided insight into age and gender related differences. This information is warranted to efficiently guide and evaluate PA promotion. Moreover the results underline the relevance of providing opportunities for unstructured play to promote PA in young children and to promote structured sports activities to increase PA in adolescents. It also emphasizes the important contribution of active forms of commuting.

## Competing interests

The authors declare that they have no competing interests.

## Authors' contributions

BB, UM, FHS and CB contributed to the study conception and design. BB, NR, and UM collected the data and were responsible for the collaboration with the school health services and teachers. UM and NR prepared the accelerometer data for analyses. BB, LG and CB conducted statistical analyses and interpreted the data. BB and CB wrote the paper. All authors critically revised the draft versions of the manuscript, provided critical feedback and approved the final version.

## Supplementary Material

Additional file 1 **Time activity diary**. An English version of the described time activity diary. Click here for file

## References

[B1] Zimmermann MB, Gubeli C, Puntener C, Molinari L (2004). Overweight and obesity in 6–12 year old children in Switzerland. Swiss Med Wkly.

[B2] Corder K, Ekelund U, Steele RM, Wareham NJ, Brage S (2008). Assessment of physical activity in youth. J Appl Physiol.

[B3] Rowlands AV (2007). Accelerometer assessment of physical activity in children: an update. Pediatr Exerc Sci.

[B4] Sallis JF, Saelens BE (2000). Assessment of physical activity by self-report: status, limitations, and future directions. Res Q Exerc Sport.

[B5] Janz KF (1994). Validation of the CSA accelerometer for assessing children's physical activity. Med Sci Sports Exerc.

[B6] Trost SG, Ward DS, Moorehead SM, Watson PD, Riner W, Burke JR (1998). Validity of the computer science and applications (CSA) activity monitor in children. Med Sci Sports Exerc.

[B7] Jago R, Anderson CB, Baranowski T, Watson K (2005). Adolescent patterns of physical activity differences by gender, day, and time of day. Am J Prev Med.

[B8] Telford A, Salmon J, Timperio A, Crawford D (2005). Examining physical activity among 5- to 6- and 10- to 12-year-old children: The Children's Leisure Activities study. Pediatric Exercise Science.

[B9] McKenzie TL, Marshall SJ, Sallis JF, Conway TL (2000). Student activity levels, lesson context, and teacher behavior during middle school physical education. Res Q Exerc Sport.

[B10] Bringolf-Isler B, Grize L, Mader U, Ruch N, Sennhauser FH, Braun-Fahrlander C (2008). Personal and environmental factors associated with active commuting to school in Switzerland. Prev Med.

[B11] Largo RH (2001). Babyjahre Die frühkindliche Entwicklung aus biologischer Sicht.

[B12] Welk GJ (2002). Physical Activity Assessments for Health-Related Research.

[B13] Weston AT, Petosa R, Pate RR (1997). Validation of an instrument for measurement of physical activity in youth. Med Sci Sports Exerc.

[B14] MeteoSwiss. http://www.meteoschweiz.admin.ch/web/en/weather.html.

[B15] Freedson P, Pober D, Janz KF (2005). Calibration of accelerometer output for children. Med Sci Sports Exerc.

[B16] STATA (2005). Statistical Software. Book Statistical Software (Editor ed^ds), Release 9 edition City.

[B17] Kirkwood B, Stern J (2003). Essential Medical Statistics.

[B18] Sallis JF, Prochaska JJ, Taylor WC (2000). A review of correlates of physical activity of children and adolescents. Med Sci Sports Exerc.

[B19] Trost SG, Pate RR, Sallis JF, Freedson PS, Taylor WC, Dowda M, Sirard J (2002). Age and gender differences in objectively measured physical activity in youth. Med Sci Sports Exerc.

[B20] Horst K Van Der, Paw MJ, Twisk JW, Van Mechelen W (2007). A brief review on correlates of physical activity and sedentariness in youth. Med Sci Sports Exerc.

[B21] Nader PR (2003). Frequency and intensity of activity of third-grade children in physical education. Arch Pediatr Adolesc Med.

[B22] Ridgers ND, Stratton G, Fairclough SJ (2005). Assessing physical activity during recess using accelerometry. Prev Med.

[B23] Burdette HL, Whitaker RC, Daniels SR (2004). Parental report of outdoor playtime as a measure of physical activity in preschool-aged children. Arch Pediatr Adolesc Med.

[B24] Merom D, Tudor-Locke C, Bauman A, Rissel C (2006). Active commuting to school among NSW primary school children: implications for public health. Health Place.

[B25] Timperio A, Ball K, Salmon J, Roberts R, Giles-Corti B, Simmons D, Baur LA, Crawford D (2006). Personal, family, social, and environmental correlates of active commuting to school. Am J Prev Med.

[B26] Andersen RE, Crespo CJ, Bartlett SJ, Cheskin LJ, Pratt M (1998). Relationship of physical activity and television watching with body weight and level of fatness among children: results from the Third National Health and Nutrition Examination Survey. Jama.

[B27] Marshall SJ, Biddle SJ, Gorely T, Cameron N, Murdey I (2004). Relationships between media use, body fatness and physical activity in children and youth: a meta-analysis. Int J Obes Relat Metab Disord.

[B28] Barr-Anderson D, Story M, Neumark-Szainer D (2008). Longitudinal trends in television viewing and dietary intake of older adolescents and young adults: Findings from Project EYT (Eating Among Teens). 2nd ICPAPH; Amsterdam.

[B29] Duncan JS, Hopkins WG, Schofield G, Duncan EK (2008). Effects of weather on pedometer-determined physical activity in children. Med Sci Sports Exerc.

[B30] Belanger M, Gray-Donald K, O'Loughlin J, Paradis G, Hanley J (2009). Influence of weather conditions and season on physical activity in adolescents. Ann Epidemiol.

