# Supplementary material for: Assessment of intensity, prevalence and duration of everyday activities in Swiss school children: a cross-sectional analysis of accelerometer and diary data
Source: Int J Behav Nutr Phys Act. 2009 Aug 5;6:50. doi: 10.1186/1479-5868-6-50 (PMC2728706; doi:10.1186/1479-5868-6-50)
Supplement: Additional file 1 — Time activity diary. An English version of the described time activity diary. [file 1479-5868-6-50-S1.pdf]

List of activities: Nursery school/first year *1 box = 1 quarter of an hour*

Please indicate your child's activities today
(to the nearest quarter of an hour)

What did your child do today?

Activity		06:00	07:00	08:00	09:00	10:00	11:00	12:00	12:00	13:00	14:00	15:00	16:00	17:00	18:00	19:00	20:00	21:00	22:00	23:00	00:00	01:00	02:00	03:00	04:00	05:00	06:00
**General:**																											
Sleeping																											
Eating																											
**School/nursery school:**																											
Lessons/nursery school																											
Physical education																											
Recess																											
**Leisure time:**																											
Homework																											
Reading or browsing through books on his/her own																											
Playing a musical instrument or singing																											
Watching TV, videos or DVDs																											
Computer or video games, Playstation, Nintendo or the Internet																											
Other quiet activities or games (listening to music or stories, drawing, painting, handiwork or board games)																											
Relatively vigorous games with friends or siblings (playground, role-playing, hide-and-seek etc.)	indoors																										
	outdoors																										
Vigorous games, running around or ball games (not in a sports club)	indoors																										
	outdoors																										
Training, riding or ballet (in a club)	indoors																										
	outdoors																										
**Travel:**																											
By foot																											
By bicycle, scooter, inline skates or skateboard																											
By train, tram or bus																											
By car																											
**Special:**																											
Accelerometer removed (e.g. during swimming)																											
Activity difficult to classify																											

PLEASE CHECK THE INFORMATION THAT YOU HAVE PROVIDED ABOVE TO ENSURE THAT THERE ARE NO GAPS.
